# Upcycling Spent LiCoO_2_ Cathodes via Surface Spinel Reconstruction for Efficient Furfural Hydrogenation

**DOI:** 10.1002/cssc.70965

**Published:** 2026-08-22

**Authors:** Giulia Maria Itri, Tapio Salmi, Andrea Donato, Adriana Pietropaolo, Pierraffaele Barretta, Chiara Alessandrello, Claudia Triolo, Daily Rodríguez‐Padrón, Francesco Mauriello, Emilia Paone

**Affiliations:** ^1^ Dipartimento DICEAM Università degli Studi Mediterranea di Reggio Calabria Reggio Calabria Italy; ^2^ Dipartimento di Chimica Biologia e Biotecnologie Università di Perugia Perugia Italy; ^3^ Laboratory of Industrial Chemistry and Reaction Engineering (TKR) Åbo Akademi University Turku/Åbo Finland; ^4^ Dipartimento di Scienze della Salute Università “Magna Graecia” di Catanzaro Catanzaro Italy; ^5^ Dipartimento di Ingegneria Industriale Università di Padova Padova Italy

**Keywords:** cobalt‐based catalyst, furfural hydrogenation, heterogeneous catalysis, spent LCO batteries, waste‐derived catalysts

## Abstract

Spent lithium‐ion batteries represent a growing environmental challenge, yet their transition‐metal‐rich cathodes remain largely underutilized as functional materials. We report a direct upcycling strategy in which LiCoO_2_ cathodes recovered from end‐of‐life smartphones are transformed into efficient heterogeneous catalysts via a single‐step oxidative calcination (600 °C, 6 h, air). The resulting material retains a predominantly layered Li_1−*x*_CoO_2_ structure with a minor spinel‐like phase formed during treatment. This reconstructed fraction significantly enhances cobalt reducibility, introducing a low‐temperature reduction feature absent in the untreated precursor. Under 20 bar H_2_ at 180 °C, the catalyst selectively converts furfural to furfuryl alcohol at short reaction times, while prolonged reaction promotes further hydrogenation to tetrahydrofurfuryl alcohol, enabling tunable selectivity. Compared with the untreated precursor and commercial references, the calcined material shows markedly improved catalytic activity. Computational simulations reveal that the spinel‐like phase provides the most favorable adsorption and activation of furfural, identifying it as the dominant catalytic motif. Catalyst deactivation is reversible, and activity is largely restored by a simple recalcination step. These results establish spent LiCoO_2_ cathodes as readily accessible cobalt‐based catalysts for upgrading biomass‐derived platform molecules, providing a circular route that couples battery‐waste valorization with sustainable chemical manufacturing.

## Graphical abstract

Spent LiCoO_2_ batteries (LCO) are directly upcycled into efficient Co‐based catalysts via surface spinel reconstruction, turning waste into function. A minor spinel‐like phase enhances reducibility and drives selective hydrogenation of furfural to furfuryl alcohol or tetrahydrofurfuryl alcohol, offering tunable selectivity and simple regeneration.

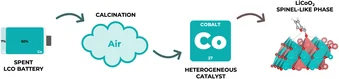

## 1 Introduction

The rapid deployment of lithium‐ion batteries (LIBs) across consumer electronics, electric mobility and grid‐scale energy storage generates an expanding stream of end‐of‐life devices that require safe collection and responsible management [1, 2]. Spent LIBs raise significant environmental and safety concerns due to reactive electrolytes, organic binders and transition‐metal‐containing cathodes, while simultaneously constituting an increasingly important secondary source of critical raw materials [3, 4, 5]. In 2022, an estimated 62 million tonnes of electronic waste (e‐waste) was generated globally, yet only 22.3% was formally collected and recycled, underscoring both the scale of the liability and the magnitude of the underutilized materials reservoir [6].

Beyond conventional battery recycling, e‐waste can be viewed as a widely distributed feedstock for heterogeneous catalysis, because it concentrates redox‐active transition metals and functional oxides in pre‐assembled solid forms that can be repurposed without full element‐by‐element separation [7, 8]. From the process technology perspective, valorization routes based on simple unit operations, particularly thermal treatment under controlled atmospheres, are inherently scalable and compatible with existing catalyst‐manufacturing infrastructure. This opportunity is amplified by the persistently low collection rates of small consumer electronics: the European Commission has reported a mobile‐phone collection rate below 5%, with approximately 700 million unused or waste handsets stored in households across the European Union, a large and poorly tapped reservoir of metal‐rich components [9].

A key challenge in LIB valorization is the chemical heterogeneity of waste streams. Layered oxides such as LiNi_*x*_Mn_*y*_Co*_z_* O_2_ (NMC) and LiNi_*x*_Co_*y*_Al*_z_* O_2_ (NCA) dominate high‐energy mobility applications, whereas LiCoO_2_ (LCO) represents compact, high‐energy‐density cells typical of portable electronics such as smartphones, laptops, and wearable devices [10, 11]. LCO is compositionally simple and cobalt‐rich: unlike multimetal cathodes, it contains a single transition metal (cobalt) within a lithium oxide framework, essentially providing a chemically well‐defined system for linking structural evolution, redox accessibility, and catalytic function [12]. Although LCO currently accounts for roughly 10% of the global LIB market by installed capacity, its relevance for circular catalysis is amplified by the specific end‐of‐life stream targeted here, namely small consumer electronics where LCO‐containing cells can represent a disproportionate fraction of disposed material [13, 14, 15, 16]. Concerning compositional simplicity, the one of Li and Co is a key advantage for direct upcycling, enabling straightforward structure‐function interpretation without the confounding effects of multi‐element cathode chemistry [17]. Because small electronic equipment is collected at low rates, LCO‐containing batteries accumulate in the disposed fraction and are therefore readily available as a cobalt‐rich feedstock for catalytic valorization [18].

Current recycling approaches increasingly emphasize “direct recycling”, which aims to preserve the electrochemical value embedded in spent cathodes by restoring crystallographic structure and lithium stoichiometry, rather than converting materials into separated element streams [19]. Despite rapid progress, most direct‐recycling routes target battery‐grade regeneration and therefore require chemistry‐specific sorting, rigorous impurity control, and additional thermal or chemical conditioning steps, increasing process complexity and energy consumption [20]. These constraints motivate alternative, lower‐barrier pathways that prioritize functional reuse of recovered electrode materials without requiring restoration to battery‐grade specifications, a concept we call “catalytic upcycling”.

Catalytic upcycling of spent electrode materials has emerged as a compelling valorization route [21, 22]. Transition‐metal oxides recovered from LIBs combine high metal content with redox flexibility and well‐established structure‐property relationships. Cobalt is particularly attractive because it accesses multiple oxidation states (Co^0^/Co^2+^/Co^3+^/Co^4+^) and can support hydrogen‐transfer chemistry under relatively mild conditions [23, 24]. In the context of biomass upgrading, cobalt‐based materials have been widely investigated as earth‐abundant alternatives to precious‐metal catalysts for hydrogenation reactions, with the activity and selectivity governed by cobalt speciation, oxygen lability, and the ease of in situ reduction to catalytically competent ensembles [25].

Furfural is a key lignocellulosic platform molecule obtained by acid‐catalyzed dehydration of pentose sugars derived from agricultural residues. Its catalytic hydrogenation yields furfuryl alcohol (FAL), a high‐value intermediate used in the production of furan resins, lubricants, and pharmaceuticals. Under more severe conditions, the ring hydrogenation produces tetrahydrofurfuryl alcohol (THFA), a biodegradable solvent with applications in agriculture and electronics [26]. The ability to tune selectivity between FAL and THFA is therefore industrially relevant and is governed by the nature and accessibility of active sites on the catalyst surface.

For spent LCO specifically, an additional structural opportunity arises from the intrinsic transformation chemistry of Li‐deficient layered oxides. Layered LCO (space group R$\bar{3}$m) can undergo oxygen‐loss‐driven reconstruction accompanied by cobalt migration, forming spinel‐like (Fd$\bar{3}$m) and, under more severe conditions, rock‐salt‐type motifs confined to near‐surface regions while the bulk remains layered [27]. Such reconstructed surface domains are known to exhibit enhanced reducibility and more labile lattice oxygen relative to well‐ordered layered environments, and have been shown to dominate the electrochemical behavior of layered cathodes even when present as a minor surface fraction [28]. By analogy, we hypothesize that thermally induced surface spinel reconstruction in spent LCO should generate redox‐active sites that are disproportionately beneficial for heterogeneous hydrogenation catalysis.

In this article, a systematic investigation of spent LCO cathodes recovered from end‐of‐life smartphone batteries as cobalt‐rich catalytic precursors is reported. Building on our previous work, which established the feasibility of directly reusing spent LiCoO_2_ as a catalyst for furfural upgrading [29], the present study advances this concept by introducing a controlled mechano‐thermal treatment that drives surface spinel reconstruction and reveals the reconstructed phase as the dominant active motif governing catalytic performance. Rather than pursuing battery‐grade regeneration, we implement a direct valorization route in which the as‐recovered layered material (l‐LCO) is subjected to a single oxidative calcination step to yield l/s‐LCO, a predominantly layered Li_1−*x*_CoO_2_ framework containing a minor, but functionally decisive, spinel‐like surface contribution. Powder X‐ray diffraction (XRD) with Rietveld refinement, micro‐Raman scattering (MRS), H_2_‐TPR, BET surface analysis, and NH_3_‐temperature‐programed desorption (NH_3_‐TPD) were used to fully characterize the structural and functional evolution induced by calcination. Catalytic performance in furfural hydrogenation is evaluated as a function of temperature, reaction time, reducing atmosphere (classical hydrogenation or transfer hydrogenation conditions), and benchmarked against commercial LiCoO_2_ and cobalt oxide references. At the same time, recyclability is demonstrated through successive catalytic experiments and oxidative regeneration. Ab‐initio molecular dynamics simulations combined with machine learning predictions are used to rationalize the superior activity of the spinel‐like surface fraction at the molecular level. Together, this approach established a simple, scalable, and scientifically grounded circular route that couples battery‐waste valorization with sustainable catalytic upgrading of biomass‐derived platform molecules.

## 2 Results and Discussion

### 2.1 Cathode Recovery and Mechano‐Thermal Treatment

**SCHEME 1:**
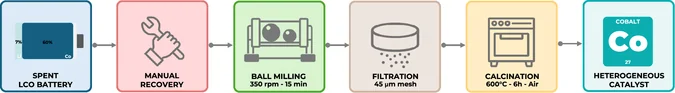
Schematic representation of the mechano‐thermal treatment of spent LCO batteries for the preparation of Co‐based heterogeneous catalysts.

The active cathode material was recovered from end‐of‐life LCO mobile phone batteries following the dry mechanical procedure in analogy with our earlier study [29]. The workflow displayed in Scheme 1 comprises manual recovery of the electrode material, followed by ball milling (350 rpm, 15 min), sieving (45 μm mesh) to remove coarse impurities, and subsequent calcination at 600 °C for 6 h in air, yielding the final Co‐based heterogeneous catalyst.

**FIGURE 1:**
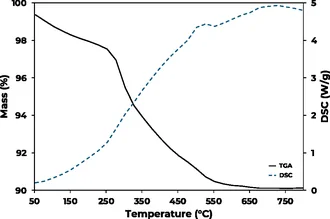
Thermogravimetric analysis (TGA) and differential scanning calorimetry (DSC) profiles of l‐LCO catalyst recorded under flowing air.

The thermal behavior of the as‐recovered cathode‐derived powder was investigated by thermogravimetric analysis coupled with differential scanning calorimetry (TGA–DSC) under flowing air, in order to reproduce the oxidative atmosphere used during catalyst preparation (Figure 1). Under oxidative conditions, the sample exhibited a progressive mass loss, with the main decrease occurring between approximately 250 and 550 °C. This mass loss can be attributed to the oxidative removal of residual organic and carbonaceous species originating from the spent cathode matrix. Above 600 °C, the mass profile became nearly stable, indicating that most thermally labile species had been removed. At higher temperatures, additional mass changes may be associated with oxygen release from the cathode‐derived oxide, while structural rearrangements of the oxide framework may occur concomitantly [30, 31]. These results support the selection of 600 °C as the calcination temperature, as this temperature provides effective oxidative cleaning of the recovered cathode powder while avoiding unnecessary high‐temperature treatment.

For comparison, the TGA–DSC profile previously recorded under flowing N_2_ is reported in Figure S1. Under inert atmosphere, the sample exhibited a gradual mass decrease without abrupt decomposition events, with a cumulative mass loss of only 3.1% and a final residue of 96.9 wt%, confirming the high thermal stability and predominantly inorganic nature of the cathode‐derived powder. The limited mass loss observed under N_2_ can be associated with the desorption of physisorbed water at low temperature, followed by the thermal decomposition and volatilization of residual organic binder and labile surface species. Compared with the inert profile, the oxidative profile displays a more pronounced mass‐loss event in the intermediate‐temperature region, consistent with combustion/oxidative removal of residual organic and carbonaceous species. This comparison confirms that the thermal behavior of the recovered powder is strongly atmosphere‐dependent and that the oxidative TGA–DSC profile is the most appropriate reference for selecting the calcination conditions.

### 2.2 Morphological and Compositional Characterization

**FIGURE 2:**
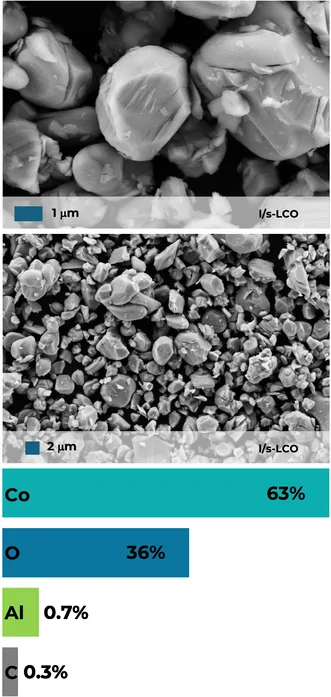
Scanning electron microscopy (SEM) images of l/s‐LCO catalyst.

The morphology of the calcined l/s‐LCO material was examined by scanning electron microscopy (SEM) at low (2.5k×) and high (10k×) magnification (Figure 2).

The micrographs reveal a polydisperse powder composed of irregular, angular particles with well‐defined crystallographic facets, consistent with the morphology expected for layered cobalt oxide phases [32, 33]. At low magnification, the sample appears as agglomerates of micron‐sized crystallites interspersed with a finer particulate fraction. At high magnification, individual particles display smooth, faceted surfaces with cleavage‐like planes and limited evidence of open mesoporosity. No extensive interparticle necking or sintering is observed, indicating that calcination at 600 °C preserves a particulate morphology rather than producing a densified compact, an important feature for maintaining reactant accessibility to active surface sites. Despite the low specific surface area, the superior catalytic performance of l/s‐LCO indicates that activity is not surface‐area‐driven but rather governed by the nature and quality of the active sites generated upon surface reconstruction.

Elemental composition was assessed by energy‐dispersive X‐ray spectroscopy (EDS) on multiple representative regions. The analyzed area is dominated by Co and O (Co: 63.72 wt%; O: 35.08 wt%), consistent with a LiCoO_2_‐type stoichiometry (note that Li is below the EDS detection limit due to its low atomic number). Only trace levels of Al were detected (0.97 wt%), attributable to current‐collector contamination during mechanical processing, and residual carbon is negligible (0.24 wt%), indicating effective removal of the organic binder residues during the oxidative calcination step [34]. The elemental composition of the material, determined by EDS analysis, revealed a clear predominance of Co and O, consistent with the expected stoichiometry of a LiCoO_2_‐type phase and in excellent agreement with values widely reported in the literature for LCO materials of analogous origin, confirming the reliability of the compositional characterization [35, 36].

**FIGURE 3:**
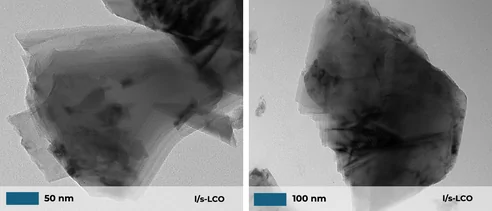
Transmission electron microscopy (TEM) images of l/s‐LCO catalyst at different magnifications.

Transmission electron microscopy (TEM) confirms platelet‐like particle morphology consistent with the layered oxide crystal structure. The particle size distribution extracted from the TEM image analysis spans the low‐micrometre range: most particles fall between approximately 3 and 10 μm with a modal size of 6–7 μm (Figure 3). A smaller fraction of sub‐micrometre fragments is also present, attributable to mechanical comminution during ball milling. Significantly, localized high‐contrast regions are observed at particle surfaces and edges, suggesting nanoscale structural or compositional heterogeneity consistent with the presence of surface‐reconstructed domains distinct from the layered bulk, a key observation corroborated by subsequent XRD and Raman analyses.

### 2.3 Structural Characterization by XRD and Micro‐Raman Spectroscopy

**FIGURE 4:**
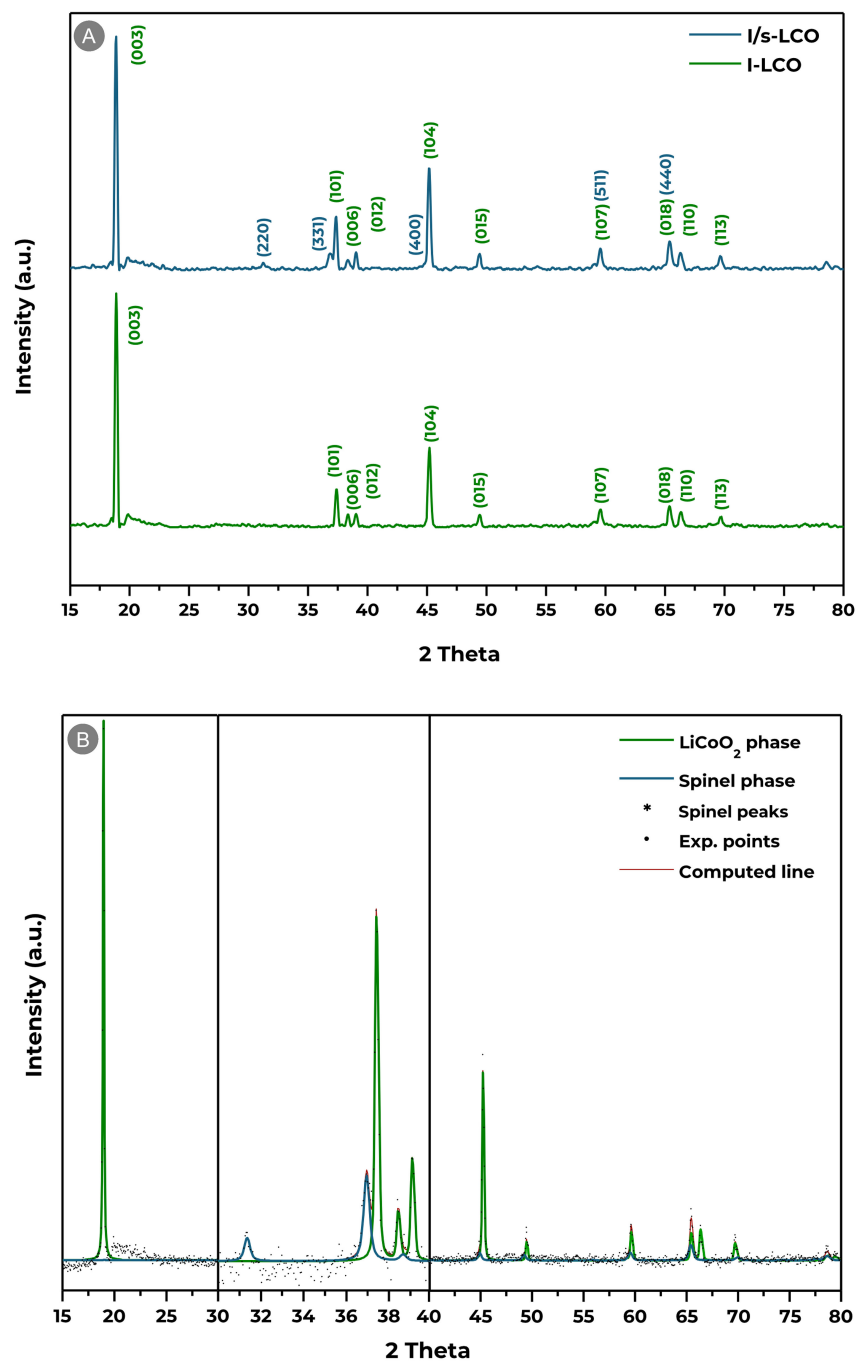
XRD analysis of l‐LCO and l/s‐LCO: (A) diffraction patterns; (B) Rietveld refinement of l/s‐LCO catalyst.

The crystalline phase composition of the calcined material was determined by powder XRD and Rietveld refinement (Figure 4).

The diffraction pattern of l/s‐LCO is dominated by the characteristic reflections of layered LiCoO_2_ with R$\bar{3}$m symmetry, including the strong low‐angle (003) reflection and the diagnostic doublet splittings of the (006)/(102) and (108)/(110) peak pairs, hallmarks of the ordered layered structure. These features confirm that the long‐range layered framework is preserved after calcination at 600 °C.

However, the calcined sample also exhibits weak additional reflections that cannot be accounted for by a single‐phase layered model. Rietveld refinement required a two‐phase model to achieve a satisfactory fit: a major layered Li_1−*x*_CoO_2_ component (R$\bar{3}$m, ≈94 wt%) together with a minor spinel‐type contribution (Fd$\bar{3}$m, ≈6 wt%). The spinel‐like phase is assigned to Li‐containing Li_1−*x*_Co_2_O_4_‐type and/or Co_3_O_4_‐type domains, consistent with the expected products of oxygen redistribution and cobalt migration in Li‐deficient layered oxides under oxidative thermal conditions. Based on this phase analysis, the calcined material is hereafter denoted l/s‐LCO, where “l” designates the layered Li_1−*x*_CoO_2_‐type majority phase and “s” the spinel‐like minority phase resolved by Rietveld refinement.

In contrast, the XRD pattern of the as‐recovered (uncalcined) powder is fully described by a single layered LiCoO_2_ phase: the (003) reflection and all characteristic doublet splittings are preserved, and no reflections attributable to a secondary spinel‐type phase are detected within the sensitivity of the measurement. This comparison establishes unambiguously that the spinel‐like contribution in l/s‐LCO is generated by oxidative calcination, rather than being a pre‐existing feature of the as‐received cathode stream.

**FIGURE 5:**
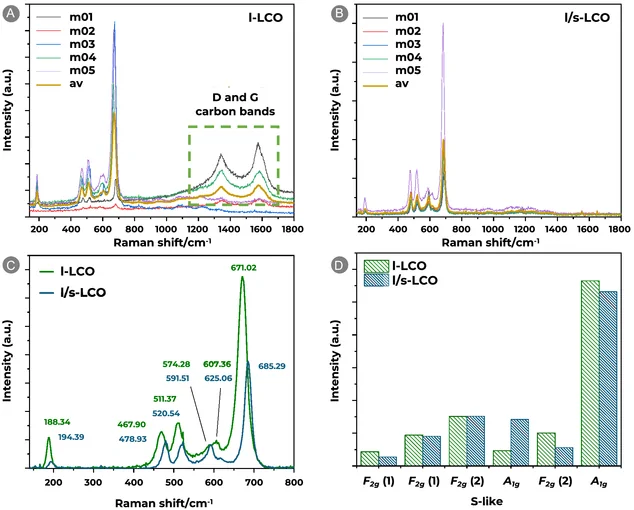
Micro‐Raman scattering (MRS) spectra: (A) spectra acquired at randomly selected positions on the l‐LCO sample surface and average spectrum; (B) spectra acquired at randomly selected positions on the l/s‐LCO sample surface and averaged spectrum; (C) comparison between the average spectra of l‐LCO and l/s‐LCO at low frequency region from 140 to 800 cm^−1^; (D) percentage of integrated area derived from the deconvolution procedure of MRS spectra using Voigt bands.

To obtain complementary information on local structural features and phase distribution, micro‐Raman scattering (MRS) analyses were conducted on both l‐LCO and l/s‐LCO powders (Figure 5).

Spectra were acquired at multiple randomly selected positions on each sample surface to assess spatial homogeneity, and final analyses were performed on averaged spectra. In the high‐frequency region (1200–1800 cm^−1^), the MRS spectra of l‐LCO display D‐band (ca. 1336 cm^−1^) and G‐band (ca. 1572 cm^−1^) features characteristic of disordered carbonaceous residues [37]. These contributions are completely absent in the spectra of l/s‐LCO, confirming that calcination at 600 °C effectively eliminates residual organic material, consistent with the EDS and TGA findings.

In the oxide‐characteristic low‐frequency region (140–800 cm^−1^), both samples exhibit bands diagnostic of multiple coexisting oxide phases. In l‐LCO, bands at 188 and 671 cm^−1^ are attributed to the F_2_g(1) and A_1_g vibrational modes of Co_3_O_4_ spinel, respectively [36]. The strongly overlapping bands in the 400–650 cm^−1^ region were resolved by Voigt‐profile deconvolution, yielding four main contributions: a band at 511 cm^−1^ assigned to the F_2_g(2) mode of Co_3_O_4_ [38], and bands at 468, 574, and 607 cm^−1^ attributed to the F_2_g(1), A_1_g, and F_2_g(2) modes of the non‐layered Li_1−*x*_Co_2_O_4_ phase (Fd$\bar{3}$m space group), respectively [39]. The Li_1−*x*_Co_2_O_4_ Fd$\bar{3}$m structure can exist as two polymorphs: an over‐lithiated cubic form with Li^+^ and Co^2+^ in octahedral sites, and a spinel‐like form with lower lithium content and Li^+^ occupying tetrahedral sites [40].

No unambiguous contribution from the layered LiCoO_2_ (R$\bar{3}$m) phase is resolved in the MRS spectra, because its characteristic O─Co─O bending (ca. 487 cm^−1^) and Co─O stretching (ca. 596 cm^−1^) modes [41] overlap spectrally with the Li_1−*x*_Co_2_O_4_ bands identified above. The presence of the layered phase is, however, unambiguously confirmed by XRD. This apparent discrepancy is consistent with the known surface sensitivity of Raman spectroscopy relative to XRD: the Raman signal is dominated by surface and near‐surface phases (where spinel‐like reconstruction is concentrated), while XRD reflects bulk‐averaged long‐range order (dominated by the layered majority).

Following calcination, the MRS spectrum of l/s‐LCO retains the same overall band pattern as l‐LCO, although slight shifts in the detected vibrational modes are observed, suggesting local structural modifications associated with lithium deficiency and the formation of non‐stoichiometric Li_1−*x*_CoO_2_ phases induced by the thermal treatment [39, 41]. The relative integrated intensities of most bands remain broadly unchanged after calcination; however, the A_1_g and F_2_g(2) modes of the Li_1−*x*_Co_2_O_4_ non‐layered phase (Figure 5D) display notable intensity variations, which are tentatively ascribed to a calcination‐induced shift in the relative abundance of the two Li_1−*x*_Co_2_O_4_ polymorphs (cubic vs. spinel‐like), combined with a possible change in the layered LiCoO_2_ background contribution in this spectral region. Overall, the XRD and MRS data provide a consistent and complementary picture of l/s‐LCO as a biphasic material in which a spinel‐like surface layer is superimposed on a layered bulk, with the surface fraction being accessible to reactants under relevant conditions for catalytic processes.

### 2.4 Surface Chemical Composition by XPS Spectroscopy

The surface composition and chemical environment of the LiCoO_2_‐derived samples were investigated by X‐ray photoelectron spectroscopy (XPS). The corresponding high‐resolution spectra are reported in Figure S4 for l‐LCO and Figure S5 for l/s‐LCO, while the main binding energies and assignments are summarized in Table 1. Survey spectra confirmed the presence of C, O, Co, and Li in both samples. Comparison of the survey spectra indicates a decrease in the relative carbon contribution after oxidative calcination, accompanied by a higher relative contribution of Co and O. This trend is consistent with the removal of carbonaceous species covering the recovered cathode surface and with the greater exposure of the underlying cobalt–oxygen environments [42].

**TABLE 1:** Main XPS binding energies of the C 1s, O 1s, Co 2p, and Li 1s core levels of l‐LCO and l/s‐LCO catalyst.

Binding energy, eV								
Sample	C 1s				O 1s	Co 2p		Li 1s
	C─C/C─H	C─O	C═O	O─C═O	Lattice O^2−^	2p_3/2_	2p_1/2_	Li‐containing species
l‐LCO	284.6	285.9	287.8	289.6	529.4	779.1	793.9	55.0
l/s‐LCO	284.6	285.7	287.1	289.0	529.0	779.4	795.5	54.8

All binding energies were charge‐corrected by setting the hydrocarbon C─C/C─H component of the C 1s spectrum to 284.6 eV. The C 1s spectra contain additional contributions associated with C─O, C═O, and O─C═O environments. The high‐binding‐energy O─C═O component is compatible with carbonate‐containing surface species, including Li_2_CO_3_. After calcination, the lower overall carbon contribution and the modified distribution of oxygen‐containing carbon species indicate substantial cleaning of the recovered cathode surface. This interpretation is consistent with the disappearance of the carbon‐related D and G bands in the Raman spectrum of l/s‐LCO and with the low residual carbon content detected by EDS.

The O 1s envelopes include a lower‐binding‐energy contribution assigned to lattice O^2−^ and higher‐binding‐energy contributions associated with carbonate‐containing, hydroxylated, and adsorbed surface oxygen species. The variation in their relative contributions after calcination indicates a modification of the surface oxygen environment. These changes are consistent with the removal of surface contaminants and with a reorganization of the near‐surface Li─Co─O framework during the oxidative treatment.

The Co 2*p* spectra exhibit the characteristic Co 2p_3/2_ and Co 2p_1/2_ spin–orbit regions together with the associated satellite structure. The overall line shape is consistent with a predominantly Co^3+^‐rich cobalt–oxygen environment in both samples, indicating that oxidative calcination does not induce a major change in the average cobalt oxidation state detected by XPS. At the same time, the increased relative contribution of cobalt in the calcined sample is consistent with a greater exposure of Co─O surface environments following the removal of carbonaceous species.

In the 52–66 eV region, the weak Li 1s contribution partially overlaps with the more intense Co 3p signal. The presence of the Li 1s component confirms that lithium‐containing species remain at the surface of both materials. Although this region does not allow a detailed distinction among the different Li‐containing environments, the combined evolution of the Li 1s/Co 3p, O 1s, and Co 2p regions is consistent with a calcination‐induced modification of the near‐surface Li─Co─O structure.

Overall, XPS shows that oxidative calcination cleans the surface of the recovered cathode material and modifies the relative exposure and chemical environment of cobalt–oxygen and lithium–oxygen species. These surface‐sensitive observations complement the XRD and Raman results and are consistent with the calcination‐induced reconstruction proposed for l/s‐LCO.

### 2.5 Redox Properties: Temperature‐Programed Reduction with Hydrogen

**FIGURE 6:**
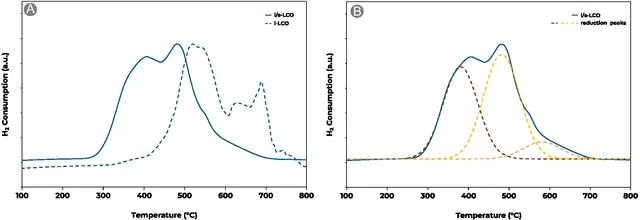
Temperature‐programed reduction (TPR) profiles: (A) TPR curves of l‐LCO and l/s‐LCO catalysts; (B) deconvoluted TPR profile of the l/s‐LCO catalyst.

The redox accessibility of the cobalt oxide framework was probed by H_2_‐temperature‐programed reduction (H_2_‐TPR), a technique particularly sensitive to near‐surface reducible motifs and lattice oxygen lability (Figure 6).

The uncalcined layered material (l‐LCO) exhibits a dominant high‐temperature reduction feature centered at approximately 520 °C, preceded by a gradual onset and followed by shoulders at ca. 620 and 690 °C. This stepwise, high‐temperature reduction profile is characteristic of cobalt in a well‐ordered layered oxide lattice, where strong Co─O bonds and limited oxygen mobility impose high kinetic barriers to reduction [43].

In sharp contrast, the calcined material (l/s‐LCO) displays a pronounced shift of reducibility toward a lower temperature. A distinct low‐temperature reduction event appears at approximately 380 °C, accompanied by an additional feature near 470 °C, indicating that a fraction of cobalt in l/s‐LCO is substantially more accessible to reduction than in the layered precursor [44, 45]. Crucially, this transformation occurs while XRD remains dominated by layered reflections and Rietveld refinement indicates only ≈6 wt% spinel‐like phase. The TPR response therefore provides direct functional evidence that the reconstructed surface domains contribute disproportionately to the overall reduction behavior relative to their bulk‐phase abundance. This is physically reasonable because H_2_‐TPR is governed by the H_2_ dissociation kinetics and O removal at accessible surface sites, rather than by bulk‐averaged long‐range order.

The low‐temperature reduction signature at 380 °C is mechanistically consistent with the formation of spinel‐like Li_1−*x*_Co_2_O_4_ and/or Co_3_O_4_‐type environments in which cation coordination environments and oxygen sublattice geometries differ from layered Co^3+^ sites, facilitating stepwise Co^3+^ → Co^2+^ → Co^0^ reduction at lower temperature [46]. Additionally, the end‐of‐life origin of l‐LCO implies partial delithiation (Li_1−*x*_CoO_2_, *x* > 0), and oxidative calcination can further amplify the near‐surface Li deficiency while promoting cobalt migration, generating Co^4+^‐rich or vacancy‐rich motifs that are intrinsically more facile to reduce. The surface enrichment of these reconstructed domains, suggested by the Raman analysis, accounts for why such a minor bulk fraction yields a prominent low‐temperature TPR feature.

Collectively, the H_2_‐TPR results indicate that oxidative calcination converts l‐LCO into a more readily reducible precursor under H_2_, as shown by the lower‐temperature reduction features compared with the uncalcined layered material. These features highlight the enhanced redox accessibility of l/s‐LCO. In this context, H_2_‐TPR is used as a comparative descriptor of the redox properties of the catalysts, while the role of pre‐reduction was further evaluated through catalytic tests using a pre‐reduced l/s‐LCO sample.

As shown in Figure S2, the oxidized l/s‐LCO catalyst outperformed the pre‐reduced sample under the standard reaction conditions, indicating that prior reduction is not beneficial for furfural hydrogenation. The reaction time effect experiments with the pre‐reduced catalyst show that furfural conversion increases with reaction time, while FAL remains the main product (Figure S3). The effect of reaction temperature reported for both systems in Table S1 also confirms that the pre‐reduced material does not provide an advantage over the oxidized l/s‐LCO catalyst. These results indicate that the superior performance of l/s‐LCO is related to the calcination‐derived oxidized/reconstructed surface state, while H_2_‐TPR should be considered as a descriptor of redox accessibility rather than as direct proof of in situ reduction during catalysis. The absence of a required pre‐reduction step also supports the practical simplicity of the proposed upcycling strategy.

### 2.6 Textural Properties and Surface Acidity

The textural properties and surface acidity of all investigated materials are summarized in Table 2.

**TABLE 2:** Textural properties and surface acidity of l‐LCO, l/s‐LCO and commercial cobalt oxide catalysts determined by N_2_ physisorption (BET surface area and pore volume from DFT and BJH models) and total acidity measured by NH_3_‐TPD analysis.

Sample	**SA**_**BET**_ **,** **m** ^**2**^ **/g**	**V**_**DFT**_ **,** **cm** ^**3**^ **/g**	**V_BJH_,** **cm** ^**3**^ **/g**	**Total Acidity,** **μmol/g**
l‐LCO	2	0.001	0.01	0.1
l/s‐LCO	7	0.003	0.02	2
Co_3_O_4_ comm	7	0.003	0.05	—
LiCoO_2_ comm	2	0.001	0.02	—

All samples exhibited relatively low specific surface areas (SA_BET_) and limited pore volumes, which is consistent with the crystalline, dense nature of cobalt oxide phases derived from solid‐state cathode precursors. The oxidative calcination treatment did not produce significant changes in textural parameters related to the uncalcined precursor, indicating that the 600 °C treatment preserves the macroscopic particle morphology without introducing substantial mesoporosity or causing interparticle sintering, in agreement with the SEM observations.

In contrast, a difference emerges in surface acidity. NH_3_‐temperature‐programed desorption (NH_3_‐TPD) revealed that l/s‐LCO exhibits a significantly higher total surface acidity than the uncalcined l‐LCO and all commercial oxide references. This increase in the surface acid site density is attributed to the formation of coordinatively unsaturated Co surface sites and Li‐vacancy‐associated defects generated during calcination, which can act as Lewis acid sites capable of coordinating furfural through its carbonyl oxygen, a key elementary step in carbonyl hydrogenation. The increased surface acidity of l/s‐LCO may contribute to furfural activation by promoting coordination of the carbonyl group through Lewis acidic surface sites. However, the catalytic trends correlate more closely with the redox properties revealed by H_2_‐TPR, suggesting that surface acidity plays a secondary, complementary role relative to cobalt reducibility.

### 2.7 Catalytic Performance in Furfural Hydrogenation

**SCHEME 2:**
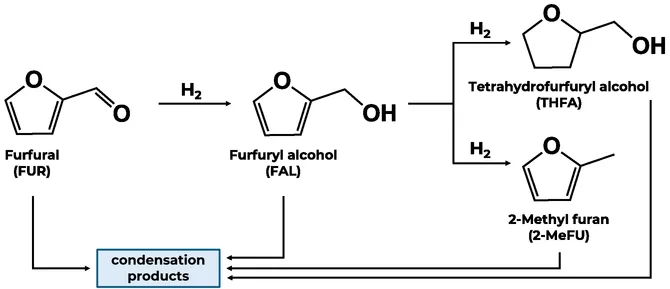
Reaction pathways for furfural (FUR) hydrogenation to furfuryl alcohol (FAL) and secondary products tetrahydrofurfuryl alcohol (THFA) and 2‐methylfuran (2MeFUR).

Catalytic hydrogenation of furfural on cobalt‐based catalysts proceeds via a consecutive reaction pathway in which the carbonyl group is first reduced to yield furfuryl alcohol (FAL) as the primary product [47]. Under more severe conditions (e.g., higher temperatures, longer reaction times, or higher H_2_ pressures), the furan ring is subsequently hydrogenated to produce THFA. This sequential network is summarized in Scheme 2. The selectivity control between FAL (a versatile resin precursor and pharmaceutical building block) and THFA (a biodegradable green solvent) is therefore a primary challenge in the catalyst design for this transformation.

**FIGURE 7:**
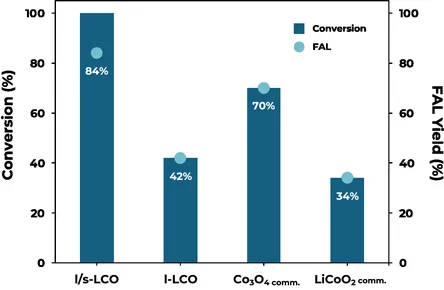
Catalytic performance comparison in furfural hydrogenation on l/s‐LCO, l‐LCO, commercial Co_3_O_4_, and commercial LiCoO_2_ catalysts (reaction conditions: 40 mL of FUR 0.1 M in 2‐propanol; catalyst/substrate molar ratio of 1:100; H_2_ pressure, 20 bar; 500 rpm; 6 h, 180 °C).

Figure 7 compares the catalytic performance of l/s‐LCO with that of the uncalcined precursor l‐LCO and the commercial reference materials Co_3_O_4_ and LiCoO_2_ under identical conditions (180 °C, 20 bar H_2_, 6 h). All catalysts were tested at identical cobalt loading, ensuring a direct comparison of intrinsic activity. l/s‐LCO achieved complete furfural conversion (100%) and clearly outperformed l‐LCO and commercial LiCoO_2_. The superior activity of l/s‐LCO relative to Co_3_O_4_ is particularly significant: it demonstrates that the catalytic enhancement cannot be attributed simply to the presence of a bulk cobalt oxide phase but arises from the specific structural reconstruction induced by calcination, namely the formation of spinel‐like surface domains within a predominantly layered matrix. To provide a broader context for the catalytic performance of l/s‐LCO, a comparison with representative Co‐based and Co‐containing catalysts reported for furfural hydrogenation is provided in Table 3. This benchmarking is intended to contextualize the activity of l/s‐LCO rather than to claim superiority over optimized state‐of‐the‐art Co‐based systems. In this regard, the significance of l/s‐LCO lies in its direct derivation from spent LiCoO_2_ electrodes, its simple preparation through oxidative calcination, and its noble‐metal‐free composition. This conclusion is reinforced by the H_2_‐TPR data, which confirm that l/s‐LCO is far more reducible than l‐LCO, and by DFT simulations showing that the spinel‐like Li_1−*x*_Co_2_O_4_ surface provides optimal furfural adsorption.

**TABLE 3:** Catalysts and reaction conditions used for furfural hydrogenation over representative Co‐based systems.

Catalyst	Conditions	**Conversion,** **%**	**FAL selectivity,** **%**	References
Co/SBA‐15	150 °C, 20 bar H_2_, 1.5 h	>95	96	[48]
Co‐N‐C@F127_0.3_	140 °C, 10 bar H_2_, 2 h	97.2	92.5	[49]
CuCo/MgO	100 °C, 20 bar H_2_, 1 h	100	99.4	[50]
CoO_*x*_/Nb_2_O_5_	70 °C, 20 bar H_2_, 4 h	99.5	96.2	[51]
l/s‐LCO	180 °C, 20 bar H_2_, 6 h	100	84	This work

**FIGURE 8:**
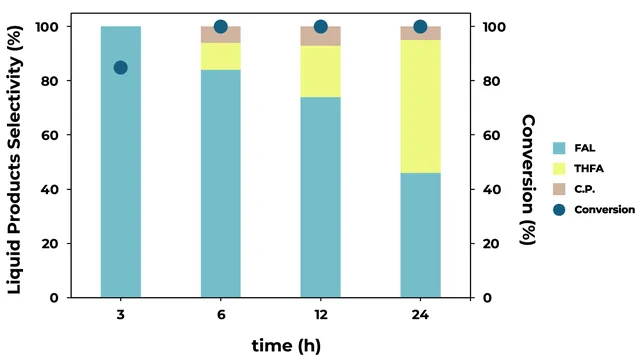
Effect of reaction time on the hydrogenation of furfural promoted by the heterogeneous l/s‐LCO catalyst (reaction conditions: 40 mL of FUR 0.1 M in 2‐propanol; catalyst/substrate molar ratio of 1:100; H_2_ pressure, 20 bar; 500 rpm; 180 °C).

The effect of reaction time on the furfural conversion and product distribution was investigated at 180 °C over 3, 6, 12, and 24 h (Figure 8). Furfural conversion increases rapidly during the initial reaction period and reaches 100% after 6 h, remaining constant at longer times. The product selectivity evolves systematically with time: at short reaction times (3 h), the system exhibited high selectivity toward FAL (>90%), consistent with rapid carbonyl hydrogenation. As the reaction time increases, the consecutive ring hydrogenation of FAL becomes significant, and selectivity progressively shifts toward THFA, which reaches approximately 50% at 24 h. This kinetic evolution is fully consistent with a consecutive FAL to THFA pathway, in which FAL is a primary intermediate that undergoes further hydrogenation once the initial carbonyl reduction is complete. The ability to tune the product distribution by reaction time control is a practical advantage of this catalytic system.

**FIGURE 9:**
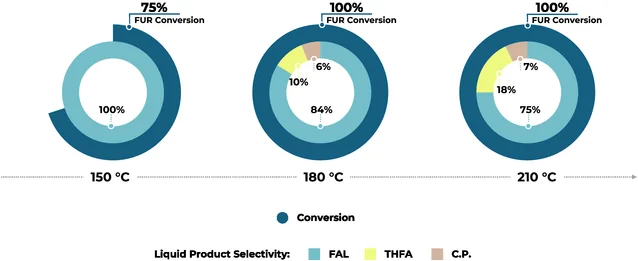
Effect of reaction temperature on the hydrogenation of furfural promoted by the heterogeneous l/s‐LCO catalyst (reaction conditions: 40 mL of FUR 0.1 M in 2‐propanol; catalyst/substrate molar ratio of 1:100; H_2_ pressure, 20 bar; 500 rpm; 6 h).

The influence of temperature was investigated at 150–210 °C (Figure 9). Furfural conversion increased with increasing reaction temperature, from 75% at 150 °C to quantitative conversion at 180 °C, and remained unchanged upon further increasing the temperature to 210 °C. The product selectivity is strongly temperature‐dependent: at 150 °C, the reaction is exclusively selective toward FAL, indicating that the carbonyl hydrogenation is kinetically preferred and the ring hydrogenation is negligible at this temperature. At 180 °C, the consecutive THFA formation becomes measurable, accompanied by a minor fraction (6%) of condensation by‐products. At 210 °C, the FAL selectivity decreases to approximately 75%, THFA reaches 18%, and secondary condensation products increase slightly to 7%. These observations confirm that the ring hydrogenation and the side reactions have higher apparent activation energies than the carbonyl hydrogenation and become competitive only at elevated temperatures. Operating at 180 °C therefore provides an optimal balance between complete furfural conversion and high FAL selectivity for applications where FAL is the target product.

**FIGURE 10:**
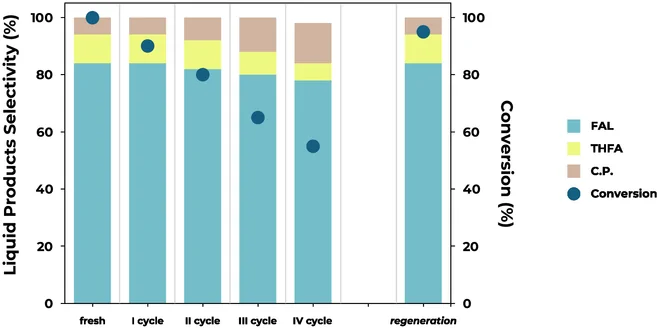
Recycling and regeneration experiments of the l/s‐LCO catalyst (reaction conditions: 40 mL of FUR 0.1 M in 2‐propanol; catalyst/substrate molar ratio of 1:100; H_2_ pressure, 20 bar; 500 rpm; 6 h, 180 °C).

The recyclability of l/s‐LCO was assessed through consecutive catalytic experiments under standard conditions (180 °C, 20 bar H_2_, 6 h), followed by a catalyst regeneration step (Figure 10). Furfural conversion decreased gradually over successive cycles, indicating progressive catalyst deactivation, likely arising from surface coke deposition and/or partial reduction of surface cobalt sites under reaction conditions. Importantly, the FAL selectivity remained constant at 100% throughout all cycles, demonstrating that the intrinsic chemoselectivity of the catalyst, which is determined by the nature of the active sites, is preserved even as overall activity declines.

After the catalyst recovery by filtration, a second oxidative calcination (600 °C, 6 h, air) was applied. This regeneration treatment restored the catalytic activity to values close to the initial performance, demonstrating that the catalyst deactivation is largely reversible and that the l/s‐LCO active‐phase structure is recovered upon re‐oxidation. The regeneration mechanism is consistent with the combustion of surface carbonaceous deposits on the catalyst surface and re‐oxidation of partially reduced cobalt sites to the active Co^3+^/spinel‐like state. The same redox‐accessible Co─O surface that contributes to the enhanced hydrogenation activity may also be more susceptible to partial reduction and/or restructuring under reaction conditions, which could contribute to the observed stability limitations. Therefore, the structural and surface characterization of the as‐prepared l/s‐LCO catalyst should be considered as representative of the initial catalyst state, while the actual surface structure under working conditions may evolve during hydrogenation. Further post‐reaction and operando characterization studies will be required to fully establish the dynamic structure–performance relationship of this catalyst system. Although oxidative regeneration restores a significant fraction of the catalytic performance, the activity decrease observed during consecutive recycling tests indicates that further optimization of catalyst stability is required before practical implementation. In this regard, the present work demonstrates the feasibility of converting spent LiCoO_2_ electrodes into active hydrogenation catalysts, while identifying long‐term stability as a key target for future catalyst design. Furthermore, in order to assess the heterogeneous nature of the catalytic process, the reaction mixture was filtered hot after 3 h and the resulting filtrate was allowed to react further under identical conditions. No additional conversion was observed, confirming that no catalytically active species leached into solution and that the activity is attributable to the solid catalyst.

### 2.8 Mechanistic Insights From Ab‐Initio Simulations

To rationalize the superior catalytic performance of l/s‐LCO on the molecular level, periodic ab initio molecular dynamics (AIMD) simulations were performed in the framework of parallel‐bias metadynamics (PBMetaD) [52] on representative surface models of the four cobalt oxide phases relevant to the catalytic system: layered Li_1−*x*_CoO_2_ (R$\bar{3}$m), spinel‐like Li_1−*x*_Co_2_O_4_ (Fd$\bar{3}$m), spinel Co_3_O_4_ (Fd$\bar{3}$m), and rock‐salt CoO. The use of metadynamics enables efficient sampling of the free‐energy landscape of furfural–surface adducts, overcoming the timescale limitations of conventional AIMD and capturing thermally accessible configurations under near‐reaction conditions. Adsorption energies were subsequently predicted from the sampled phase‐space basins using machine‐learning (ML) models within the Universal Model for Atoms (UMA) framework [53].

**FIGURE 11:**
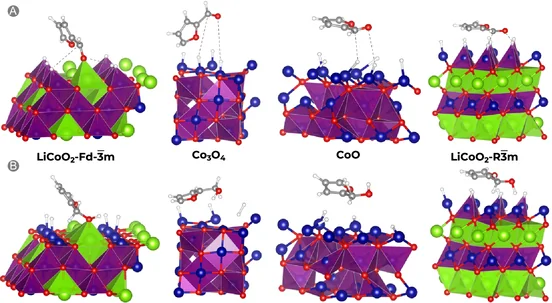
(A) Snapshots of the adsorbed furfural‐catalysts. (B) Snapshots of the adsorbed furfuryl alcohol‐catalysts. All the models are reported in ball and stick representation. Carbons are shown in silver, hydrogens in white, oxygens in red, cobalt in blue, lithium in green. The two reaction coordinates identifying the furfuryl alcohol bond formation are highlighted by dashed lines.

The C═O···H distances between the furfural carbonyl oxygen and carbon atoms and the nearest surface hydrogen atoms were employed as collective variables (reaction coordinates) to map the catalytic hydrogenation pathway. Representative snapshots of the lowest free‐energy furfural–surface and furfuryl alcohol–surface adducts are reported in Figure 11. Across all the surfaces examined, C═O···H distances spanning 2.20–4.45 Å were sampled. The most favorable geometry identifies a distance approaching 3.09 Å. It is found exclusively on the spinel‐like Li_1−*x*_Co_2_O_4_ (Fd$\bar{3}$m) surface, positioning the carbonyl bond optimally for a nucleophilic hydride attack. On the contrary, the layered Li_1−*x*_CoO_2_ (R$\bar{3}$m) surface exhibits steric crowding, with C═O···H distances approaching 2.30 Å that are indicative of repulsive steric interactions unfavorable to productive adsorption. The spinel Co_3_O_4_ (Fd$\bar{3}$m) and rock‐salt CoO surfaces display distances of 4.39 and 3.44 Å, respectively, reflecting weaker and geometrically less optimal substrate–surface interactions.

These geometric trends are quantitatively corroborated by the UMA‐predicted adsorption energies reported in Table 4. The spinel‐like Li_1−*x*_Co_2_O_4_ (Fd$\bar{3}$m) surface exhibits by far the most exothermic furfural adsorption energy (E_ads_ = −0.65 eV), followed by Co_3_O_4_ (−0.11 eV) and CoO (−0.03 eV). Strikingly, the layered Li_1−*x*_CoO_2_ (R$\bar{3}$m) surface yields a positive adsorption energy (+1.03 eV), indicating thermodynamically unfavorable binding, fully consistent with the sterically crowded C═O···H geometry identified above and with the low catalytic activity of l‐LCO observed experimentally.

**TABLE 4:** Adsorption energy values (eV) predicted for the furfural adsorbed on the surface catalysts.

Surface catalyst	Catalyst	Furfural	Catalyst‐furfural	E_Ads
CoO	−296.354	−69.534	−365.918	−0.03
Co_3_O_4_	−334.313	−68.261	−402.681	−0.11
LiCoO_2_ Fd‐$\bar{3}$m	−485.265	−69.265	−555.183	−0.65
LiCoO_2_ R‐$\bar{3}$m	−349.109	−69.251	−417.334	+1.03

Inspection of the adsorption configurations in Figure 11 reveals a clear structure–activity relationship: favorable hydrogenation is associated with a predominantly perpendicular orientation of the furfural molecule toward the catalyst surface, which maximizes carbonyl–surface orbital overlap and minimizes steric repulsion with neighboring lattice atoms. On the less active surfaces (R$\bar{3}$m and CoO), furfural adopts a flat‐lying, parallel orientation that precludes efficient C═O activation. On the spinel‐like Li_1−*x*_Co_2_O_4_ (Fd$\bar{3}$m) surface, the perpendicular adsorption mode is further stabilized by a cooperative bimetallic interaction: the carbonyl oxygen coordinates simultaneously to a surface Co^3+^ site and to an adjacent Li^+^ ion, the latter acting as a Lewis acid that polarizes the C═O bond and lowers the barrier for hydride transfer. This synergistic Co^3+^/Li^+^ activation mechanism is unique to the spinel‐like phase and is absent in both Co_3_O_4_, which lacks structural Li, and layered LiCoO_2_, where Li^+^ is not surface‐exposed in a geometry compatible with cooperative carbonyl coordination.

These computational findings are in excellent quantitative and mechanistic agreement with the experimental results: the activity ranking Li_1−*x*_Co_2_O_4_ (Fd$\bar{3}$m) >> Co_3_O_4_ > CoO >> LiCoO_2_ (Fd$\bar{3}$m) predicted by the adsorption energies mirrors the experimentally observed catalytic performance of l/s‐LCO, commercial Co_3_O_4_, and l‐LCO under identical reaction conditions. Critically, the calculations provide a molecular‐level explanation for why a minority spinel‐like surface fraction (≈6 wt% by Rietveld refinement) can dominate the overall catalytic activity of l/s‐LCO: the thermodynamic and geometric superiority of the Fd$\bar{3}$m surface for furfural activation means that even a small density of these sites can outcompete the far more abundant yet intrinsically less active layered Li_1−*x*_CoO_2_ surface. Together with the H_2_‐TPR evidence for enhanced reducibility and the NH_3_‐TPD evidence for increased surface acidity, the AIMD/UMA calculations establish a self‐consistent mechanistic picture in which the thermally induced layered‐to‐spinel surface reconstruction is the decisive structural transformation that confers on l/s‐LCO its superior catalytic function.

### 2.9 Hydrogenation Reaction Mechanism

**FIGURE 12:**
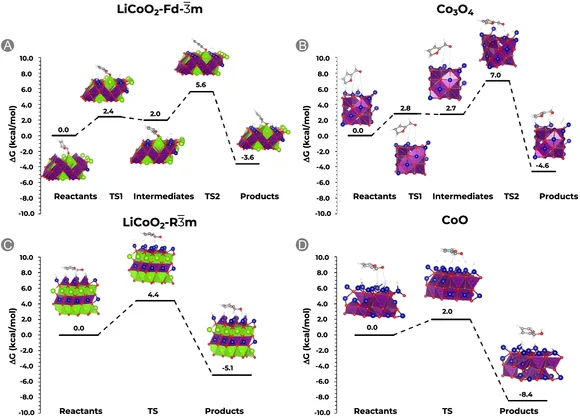
Reconstructed free energy profiles obtained from parallel bias metadynamics simulations of the hydrogenation reaction mechanism on (A) spinel like form of Li_1–x_CoO_2_ (Fd$\bar{3}$m), (B) spinel Co_3_O_4_, (C) layered form of LiCoO_2_ (R$\bar{3}$m), and (D) rock salt CoO. Snapshots of free‐energy minima, transition state (TS) and and intermediate basins are reported with the relative energetics expressed in kcal/mol.

Analysis of the parallel bias metadynamics trajectories allowed the reconstruction of the free energy profiles for furfural hydrogenation on the four investigated catalysts, providing a mechanistic explanation for their different catalytic performance (Figure 12). For each system, the free energy of the reactant minimum was taken as reference, and the relative energies of the transition states, intermediates, and products were determined from the reconstructed free energy surfaces. The spinel‐like form of Li_1−*x*_CoO_2_ (Fd$\bar{3}$m) and the spinel Co_3_O_4_ surfaces exhibit a two‐step reaction mechanism characterized by two distinct transition states separated by an intermediate. In contrast, the layered form of LiCoO_2_ (R$\bar{3}$m) and the rock salt CoO follow a single‐step reaction mechanism involving only one transition state.

For the spinel‐like form of Li_1−*x*_CoO_2_, the reactant state is stabilized by the interaction between the carbonyl oxygen of furfural and a Li^+^ ion of the surface, in agreement with the strongest adsorption energy predicted by UMA calculations. The hydrogen transfer requires a first energy barrier of 2.4 kcal/mol, reaching a metastable intermediate located at 2.0 kcal/mol. The process continues with a second barrier of 3.6 kcal/mol, and proceeds to form the product with a final free energy of −3.6 kcal/mol. The spinel Co_3_O_4_ exhibits a similar behavior. The first transition state is located at 2.8 kcal/mol, followed by a nearly isoenergetic intermediate at 2.7 kcal/mol. The second transition state represents the RDS with an energy barrier of 4.3 kcal/mol, while the products are stabilized by 4.6 kcal/mol with respect to the reactants. The layered LiCoO_2_ displays a one‐step hydrogenation mechanism with a single transition state located at 4.4 kcal/mol, leading directly to products at −5.1 kcal/mol. The rock salt CoO presents a small energy barrier of 2.0 kcal/mol. Although CoO exhibit the lowest activation energy, its weaker adsorption as predicted by UMA calculations, is expected to reduce the surface concentration of furfural, limiting the catalytic efficiency. In contrast, Li_1−*x*_CoO_2_ (Fd$\bar{3}$m) combines the most favorable adsorption strength with a reaction pathway involving two accessible energy barriers, providing an optimal balance between reactant stabilization and kinetics. LiCoO_2_ (R$\bar{3}$m) presents a higher energy barrier than the spinel‐like form. This computational evidence is fully consistent with the experimental observations where the Li_1−*x*_CoO_2_ (Fd$\bar{3}$m) exhibits the highest furfural conversion and the highest furfuryl alcohol yield.

## 3 Conclusions

We have demonstrated that spent LiCoO_2_ cathodes recovered from end‐of‐life portable electronic devices can be directly upcycled into highly efficient heterogeneous catalysts for biomass‐derived furfural hydrogenation through a single‐step oxidative calcination (600 °C, 6 h, flowing air). The key findings of this study are listed below:

Oxidative calcination converts the as‐recovered layered Li_1−*x*_CoO_2_ (l‐LCO) into a biphasic l/s‐LCO material retaining ≈94 wt% layered phase while generating ≈6 wt% spinel‐like Li_1−*x*_Co_2_O_4_‐type domains at particle surfaces, as quantified by Rietveld refinement of XRD data and confirmed by micro‐Raman spectroscopy. Despite its minor bulk abundance, this reconstructed surface fraction exerts a disproportionate functional impact: H_2_‐TPR revealed a prominent low‐temperature reduction feature at ≈380 °C, absent in l‐LCO, indicating enhanced redox accessibility, while NH_3_‐TPD evidenced a higher density of surface acid sites. XPS analysis further showed that oxidative calcination decreases the contribution of carbonaceous and carbonate‐like surface species and increases the exposure of Co─O environments, while cobalt remains predominantly in a trivalent state. Together, these observations are consistent with the superior catalytic performance of l/s‐LCO relative to the benchmark catalysts investigated.

In catalytic experiments, l/s‐LCO achieved complete furfural conversion at 180 °C and 20 bar H_2_ within 6 h, with a selectivity of furfuryl alcohol (FAL) and THFA controllable by the reaction time and temperature: short contact times and moderate temperatures (150–180 °C) strongly favor FAL, while prolonged reaction time or higher temperatures drive consecutive ring hydrogenation toward THFA. Catalyst deactivation in successive recycling experiments was substantially reversible, and the activity was largely restored by a simple second calcination step (600 °C, 6 h, air), avoiding the need for catalyst re‐synthesis. The system also operates via catalytic transfer hydrogenation using 2‐propanol as hydrogen donor under inert atmosphere, though direct H_2_ hydrogenation remains the more efficient pathway. Periodic ab initio molecular dynamics simulations (parallel‐bias metadynamics) combined with machine‐learning adsorption energy predictions (UMA) rationalize these observations at the molecular level: the spinel‐like Li_1−*x*_Co_2_O_4_ (Fd$\bar{3}$m) surface exhibits an adsorption energy of −0.65 eV, substantially more exothermic than Co_3_O_4_ (−0.11 eV), CoO (−0.03 eV), or layered LiCoO_2_ R$\bar{3}$m (+1.03 eV), and uniquely stabilizes furfural in a perpendicular adsorption geometry through a cooperative Co^3+^/Li^+^ carbonyl activation mechanism, identifying the reconstructed minority phase as the dominant active motif.

Collectively, these results establish a scientifically grounded and practically simple circular route that repurposes a widely available battery‐waste stream as a functional catalytic material. The proposed approach requires no separation of individual elements, no re‐synthesis, and no precious metals, and is compatible with existing industrial thermal‐treatment infrastructure. This work contributes to the broader goal of developing closed‐loop strategies that couple the management of critical‐material waste with sustainable catalytic chemistry for the conversion of renewable biomass‐derived feedstocks.

## 4 Experimental Section

### 4.1 Materials and Chemicals

Lithium cobalt (III) oxide (LiCoO_2_, 97%), cobalt (II, III) oxide (Co_3_O_4_), and cobalt (II) oxide (CoO, 95%) were purchased from Thermo Fisher Scientific. 2‐Propanol (HPLC grade) was supplied by Fisher Chemical. Furfural was purchased from Sigma–Aldrich and purified by distillation prior to use. All chemicals were used as received without further purification, unless otherwise specified.

### 4.2 Catalyst Preparation Procedure

Lithium cobalt oxide (LiCoO_2_) was directly recovered from spent LIBs collected from a municipal recycling facility sited in Reggio Calabria (Italy). Only end‐of‐life mobile phone batteries based on LiCoO_2_ (LCO) cathodes were selected. Cells were manually opened, and the LiCoO_2_ cathode layer was mechanically separated by carefully scraping it from the aluminum current collector. The recovered cathode material was then sieved using a 45 μm mesh to remove residual aluminum fragments and subsequently homogenized by ball milling apparatus at 350 rpm for 15 min. A fraction of the resulting powder was calcined in static air at 600 °C for 6 h (slow cooling inside muffle furnace to RT) to obtain an oxidized cobalt‐rich material (Scheme 1).

The samples were coded as l‐LCO for the material obtained before the thermal treatment and l/s‐LCO for the material obtained after that.

### 4.3 Characterization Techniques

The thermal behavior and catalyst stability of the samples were investigated by thermogravimetric analysis coupled with differential scanning calorimetry (TGA–DSC). Measurements were carried out using a TA Instruments SDT 650 thermal analyzer. The samples were heated from 50 to 850 °C at a constant heating rate of 10 °C min^−1^ under a continuous flow of nitrogen (50 mL min^−1^). The inert atmosphere was selected to assess the intrinsic thermal stability of the cathode‐derived powder while minimizing oxidative contributions during analysis [54, 55].

Morphological features and microstructural characteristics of the samples were investigated by electron microscopy. Scanning electron microscopy coupled with energy‐dispersive X‐ray spectroscopy (SEM–EDX) was performed using a Zeiss LEO Gemini 1530 microscope equipped with a Thermo Scientific UltraDry silicon drift detector (SDD). TEM was performed using a JEOL JEM‐1400Plus. Samples were dispersed in ethanol, deposited onto copper grids, and dried prior to imaging [56].

The crystalline structure and phase composition were investigated by powder XRD. Diffraction patterns were collected at room temperature using a Bruker D2 PHASER diffractometer equipped with Ni β‐filtered Cu Kα radiation (*λ* = 1.54 Å). Data were acquired in the 2*θ* range between 10° and 80°.

Phase identification was performed by comparison with reference diffraction patterns. Lattice parameters were refined by Rietveld analysis using MAUD (version 2.992) [57, 58].

XPS analyses were carried out using a Physical Electronics VersaProbe II Scanning XPS Microprobe with a monochromatic Al Kα source under 10–7 Pa. Spectra were calibrated to the C 1s peak at 284.6 eV, with data fitting performed using PHI ACCESS ESCA‐F V6 software (Shirley background and Gauss–Lorentz profiles).

Micro‐Raman spectroscopy (MRS) was employed to assess the spatial homogeneity of the oxide in terms of phase composition. Spectra were collected at room temperature using an NTEGRA Spectra SPM NT‐MDT equipped with a 532 nm laser (power: 250 μW) by collecting spectra from several random positions [59].

The reducibility of cobalt species was investigated by temperature‐programed reduction with hydrogen (H_2_‐TPR) using a BELCAT II analyzer. This technique was employed to evaluate the reduction behavior of cobalt oxide phases and to monitor the sequential Co_3_O_4_ → CoO → Co^0^ transformations [55].

Samples were pretreated in situ under a continuous argon flow (30 sccm) by heating from room temperature to 200 °C, followed by an isothermal step at 200 °C and subsequent cooling to approximately 35 °C. The reduction step was then carried out under a 5 vol% H_2_/Ar gas mixture at a total flow rate of 30 sccm. The temperature was increased from approximately 35 to 800 °C at a constant heating rate of 10 °C min^−1^, followed by an isothermal hold of 20 min at the final temperature. Hydrogen consumption was continuously monitored as a function of temperature using a thermal conductivity detector (TCD).

Textural properties were determined by nitrogen adsorption –desorption measurements using a Micromeritics 3Flex analyzer. Prior to analysis, the samples were degassed under vacuum using a Micromeritics VacPrep 061 system, followed by an additional in situ degassing step in the analysis port. The specific surface area was calculated using the Brunauer–Emmett–Teller (BET) method [60].

The pore size distribution and pore volume were evaluated using the Barrett–Joyner–Halenda (BJH) method applied to both adsorption and desorption branches, as well as density functional theory (DFT) analysis for pore size distribution [61, 62].

The total acidity of the surface was evaluated by temperature‐programed desorption (NH_3_‐TPD) of ammonia using the BELCAT II analyzer (Microtrac MRB). Prior to ammonia adsorption, samples were activated in situ by heating to 200 °C at a rate of 10 °C min^−1^ and maintained at this temperature for 1 h under helium flow. After cooling to 50 °C under He (10 mL min^−1^), ammonia adsorption was carried out by exposing the samples to a gas mixture containing 5 vol% NH_3_ in He for 1 h. Weakly physisorbed ammonia was subsequently removed by flushing with He at 50 °C for 1 h. The desorption step was performed by heating the samples from 50 to 800 °C at 10 °C min^−1^ under a He flow of 10 mL min^−1^, while monitoring the NH_3_ desorption profile. The total amount of desorbed ammonia (CDES, mol NH_3_ g^−1^) was calculated as follows [63]:

(1) $$\text{CDES}=\frac{1}{{\text{m}}_{\text{cat}}}\frac{V_{\mathrm{He}}}{V_{m}}\frac{1}{\text{β}}\int_{T_{0}}^{T_{f}}{\text{C}}_{\text{i}}\;\text{dT}$$

In this expression, *m*_cat_ represents the catalyst amount, *V*_He_ is the volumetric flow rate of the carrier gas (mL He min^−1^), *V*_m_ is the molar volume of an ideal gas under standard conditions (L mol^−1^), *β* is the heating rate (°C min^−1^), and *C* *_i_* is the ammonia concentration measured by the TCD detector as a function of temperature. The integration was performed between the initial desorption temperature *T*_0_ and the final temperature *T*_f_.

### 4.4 Evaluation of Catalytic Performance

Catalytic hydrogenation studies were carried out in a stainless‐steel autoclave reactor (Parr Instrument, model 4848), equipped with a mechanical stirrer operating at 500 rpm. The reactor was charged with a suspension of catalyst in a 0.1 M furfural solution in 2‐propanol (catalyst/substrate molar ratio of 1:100). Prior to the reaction, the autoclave was purged and pressurized with high‐purity selected gas (Ar or H_2_). The reactor was heated to the desired temperature (150, 180, 210 °C), which was monitored by an internal thermocouple connected to the reactor controller. Reaction time was counted once the target temperature was reached. Liquid samples were collected at predetermined time intervals (0, 3, 6, 12, and 24 h) using a sampling system equipped with a cooling unit directly connected to the reactor system. For catalyst recycling experiments, the solid catalyst was recovered after each run, thoroughly washed with 2‐propanol, and reused with fresh reactants under identical reaction conditions.

### 4.5 Product Analysis

The liquid‐phase reaction products were analyzed by gas chromatography using both a gas chromatograph equipped with a flame ionization detector (GC–FID) and a gas chromatograph coupled with a mass spectrometer (GC–MS). In both cases, analyses were performed using a capillary HP‐5 column (30 m × 0.32 mm × 0.25 µm). GC–FID was employed for quantitative analysis, while GC–MS was used for product identification and confirmation of peak assignments. Furfural conversion, product selectivities and yields were calculated based on the quantitative GC–FID results. The furfural conversion (*C*%), product selectivity (*S*%), and product yield (*Y*%) were calculated as follows:

(2) $$C\%=\frac{\text{mol of reacted substrate}}{\text{mol of substrate feed}}\times 100$$

(3) $$S\%=\frac{\text{mol of specific product in liquid phase}}{\text{sum of mol of all products in liquid phase}}\times 100$$

(4) $$Y\%=\frac{\text{mol of specific product}}{\text{mol of substrate feed}}\times 100$$

### 4.6 Computational Methods

The catalytic reduction of furfural to furfuryl alcohol was predicted by comparing the adsorption mechanism of furfural on four cobalt‐based catalysts: CoO, Co_3_O_4_, and LiCoO_2_ in their Fd‐3m and R‐$\bar{3}$m crystallographic phases. Ab initio periodic molecular dynamics simulations based on parallel‐bias metadynamics [52] in the well‐tempered [64] framework were performed using the CP2K [65] code in combination with the PLUMED [66, 67] plugin, applying periodic boundary conditions to all the coordinates. The catalyst models were constructed using the crystallographic coordinates deposited in the Materials Project database (mp‐19079 for CoO, mp‐18748 for Co_3_O_4_, mp‐849273 and mp‐22526 for LiCoO_2_ Fd‐3m and R‐$\bar{3}$m phases, respectively). The Co^3+/2+^ ions in each model were hydrogenated, and one furfural molecule was adsorbed onto the catalyst surface. The hybrid Gaussian and plane waves method (GPW) as implemented in the Quickstep module of CP2K was used during the simulations. Temperature control was implemented using a velocity rescaling thermostat [68] with a target temperature of 298 K and a time constant of 50 fs. The pressure was maintained at 1.013 atm. The electronic structure properties were calculated using the PBE [69, 70] functional with the Grimme correction scheme to account for dispersion interactions. Kohn−Sham orbitals were expanded in a Gaussian basis set (DZVP‐MOLOPT for Co, O, C, H, and Li), employing norm‐conserving GTH pseudopotentials for all atoms [71]. The integration time step for the dynamic equations was set to 0.5 fs, with a total simulation length of 3.6 ps. Enhanced sampling of the reaction coordinates was achieved using a combination of well‐tempered [64] and parallel‐bias metadynamics [52] within a multiple‐walker scheme, considering two replicates for each system. Two collective variables identify the catalytic patway. The first corresponds to the distance between the carbonyl oxygen atom of furfural and a hydrogen atom, whereas the second corresponds to the distance between the carbonyl carbon atom of furfural and a second hydrogen atom involed in the reduction process. Gaussian hills with an initial height of 0.4 kcal mol^−1^ and a width of 0.1 Å were deposited with a stride of 1 fs. A time step of 1 fs and a temperature of 300 K were employed with a bias factor of 10. This strategy enabled an efficient exploration of the relevant configurational space and provided representative structures for subsequent adsorption energy calculations.

The adsorption energy of furfural on the hydride catalyst surfaces was evaluated using the Universal Molecular Assembler (UMA) calculator [72] which is based on the FAIR‐Chem [73] model and trained on the Open Catalyst 2020 (OC20) dataset [74], employing an RPBE‐level theoretical framework [75]. Adsorption energies were computed according to Equation (5).

For each system, a representative adsorbate‐furfural‐catalyst configuration was extracted from the AIMD trajectory. From this configuration, the geometries of the isolated catalyst and the adsorbed furfural molecule were extracted to compute their absolute energies with UMA, namely *E*_Cat_, *E*_Furf_, and *E*_Cat.@Furf._ for the catalyst, furfural, and the adsorbed furfural–catalyst complex, respectively.

(5) $$\begin{array}{l} \mathrm{Cat}\text{.}+\text{Furf}\text{.}\to \;\mathrm{Cat}\text{.}-\text{Furf}\text{.}\\ \;E_{\mathrm{Ads}}=E_{\mathrm{Cat}\text{.}-\text{Furf}\text{.}}-E_{\mathrm{Cat}\text{.}}-E_{\text{Furf}\text{.}} \end{array}$$

## Funding

This study was supported by Ministero dell'Università e della Ricerca (P2022N48ZC_001).

## Conflicts of Interest

The authors declare no conflicts of interest.

## Supporting information

The authors have cited additional references within the Supporting Information.

## Data Availability

The data that support the findings of this study are available from the corresponding author upon reasonable request.
