# Supplementary material for: Upcycling Spent LiCoO2 Cathodes via Surface Spinel Reconstruction for Efficient Furfural Hydrogenation
Source: ChemSusChem. 2026 Aug 22;19(16):e70965. doi: 10.1002/cssc.70965 (PMC13499250; doi:10.1002/cssc.70965)
Supplement: Supplementary file 1 [file CSSC-19-e70965-s001.pdf]

Upcycling Spent $LiCoO_2$ Cathodes via Surface Spinel Reconstruction for Efficient Furfural Hydrogenation

Giulia Maria Itri,[a,b] Tapio Salmi,[c] Andrea Donato,[a] Adriana Pietropaolo,[d] Pierraffaele Barretta,[d] Chiara Alessandrello,[a] Claudia Triolo,[a] Daily Rodríguez-Padrón,[e] Francesco Mauriello,[a] and Emilia Paone*[a]

[a] G. M. Itri, A. Donato, C. Alessandrello, C. Triolo, F. Mauriello, E. Paone
Dipartimento DICEAM
Università degli Studi Mediterranea di Reggio Calabria, Reggio Calabria, Italy
Via Rodolfo Zehender, 89122 Reggio Calabria, Italy
E-mail: emilia.paone@unirc.it

[b] G. M. Itri
Dipartimento di Chimica, Biologia e Biotecnologie
Università di Perugia
Via Elce di Sotto 8, 06123 Perugia, Italy

[c] T. Salmi
Laboratory of Industrial Chemistry and Reaction Engineering (TKR)
Åbo Akademi University
Turku/Åbo, Finland

[d] A. Pietropaolo, P. Barretta
Dipartimento di Scienze della Salute
Università "Magna Graecia" di Catanzaro
Viale Europa, 88100 Catanzaro, Italy

[e] D. Rodríguez-Padrón
Dipartimento di Ingegneria Industriale
Università di Padova
Via Marzolo 9, 35131 Padova, Italy

Supporting information for this article is given via a link at the end of the document.

WILEY-VCH

SUPPLEMENTARY INFORMATION

Supporting information contains 7 pages including 5 Figures and 1 Table.

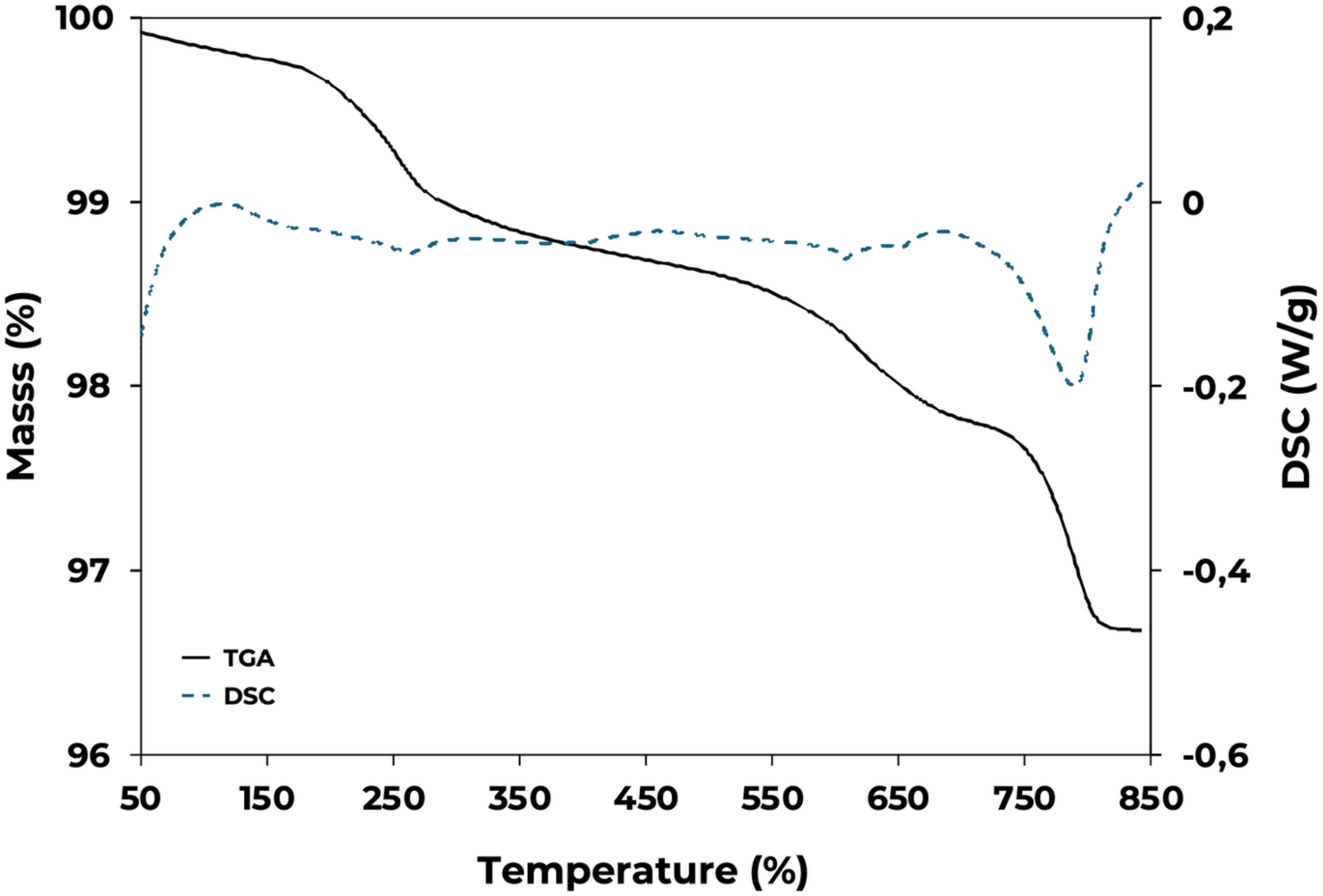

Figure S1. Thermogravimetric analysis (TGA) and differential scanning calorimetry (DSC) profiles of l/s-LCO catalyst recorded under flowing N_2.

WILEY

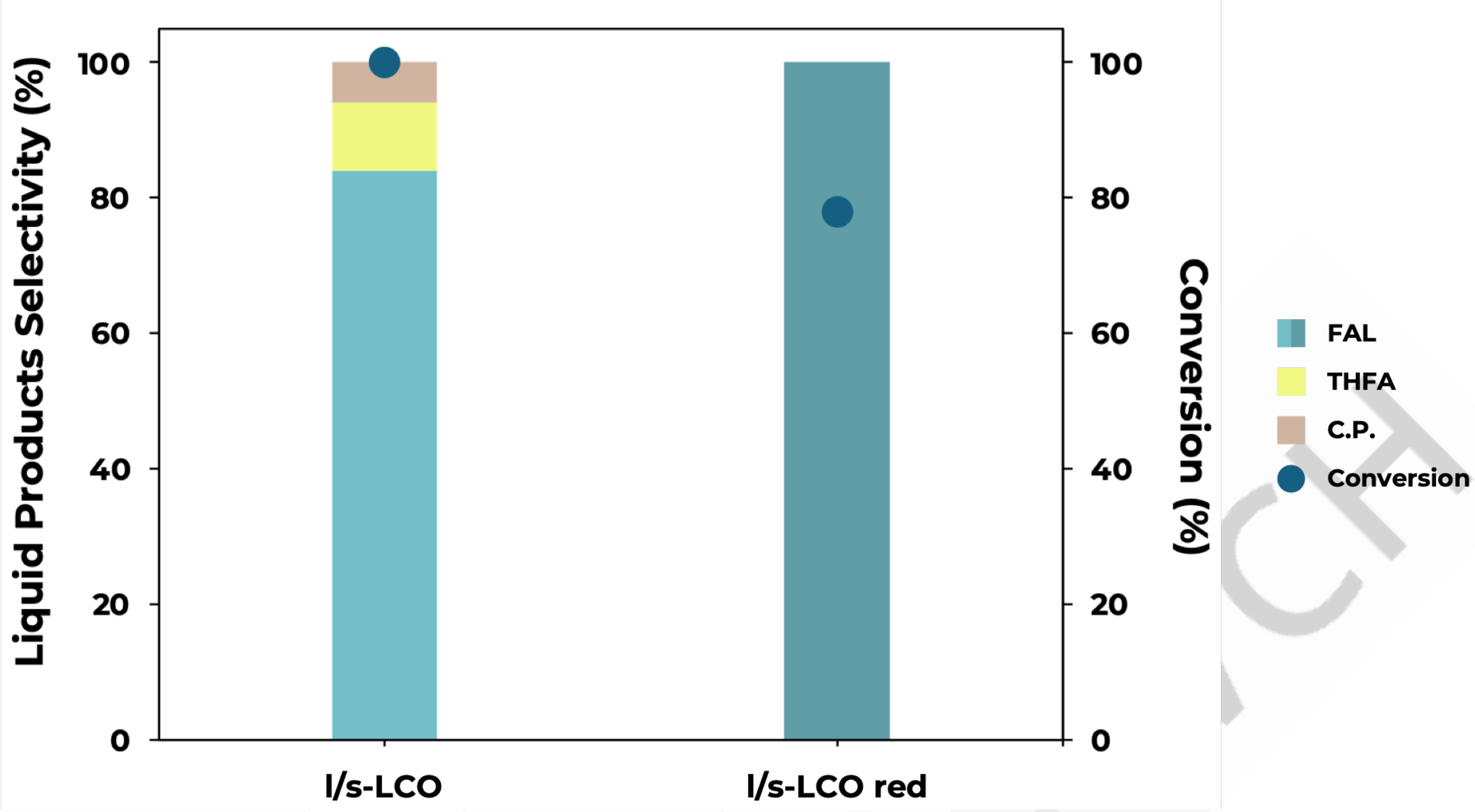

Figure S2. Comparison of the catalytic performance of I/s-LCO and pre-reduced I/s-LCO in furfural hydrogenation (reaction conditions: 40 mL of FUR 0.1 M in 2-propanol; catalyst/substrate molar ratio of 1:100; H2 pressure, 20 bar; 500 rpm; 180 °C; 6 h).

WILEY-VCH

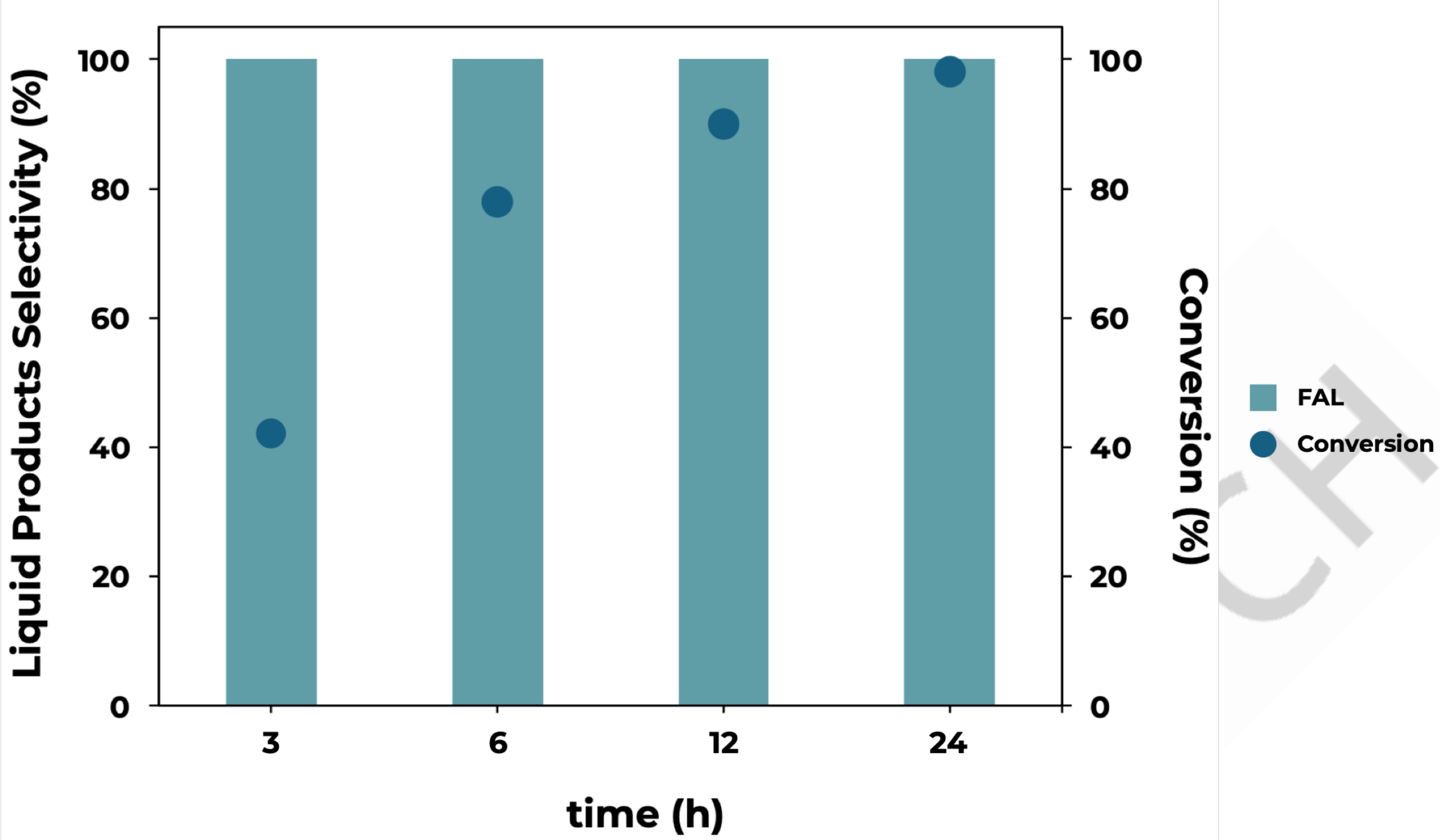

Figure S3. Effect of reaction time on furfural hydrogenation over the pre-reduced l/s-LCO catalyst (reaction conditions: 40 mL of FUR 0.1 M in 2-propanol; catalyst/substrate molar ratio of 1:100; H2 pressure, 20 bar; 500 rpm; 180 °C).

WILEY-VCH

Table S1. Effect of reaction temperature on furfural hydrogenation over l/s-LCO and pre-reduced l/s-LCO catalysts (reaction conditions: 40 mL of FUR 0.1 M in 2-propanol; catalyst/substrate molar ratio of 1:100; H2 pressure, 20 bar; 500 rpm; 6 h).

Catalyst	T (°C)	Conversion (%)	Liquid Products Selecitivity (%)		
			FAL	THFA	C.P.
l/s-LCO	150	75	100	-	-
l/s-LCO red	150	26	100	-	-
l/s-LCO	180	100	84	10	6
l/s-LCO red	180	78	100	-	-
l/s-LCO	210	100	75	18	7
l/s-LCO red	210	92	95	1	4

WILEY-VCH

WILEY-VCH

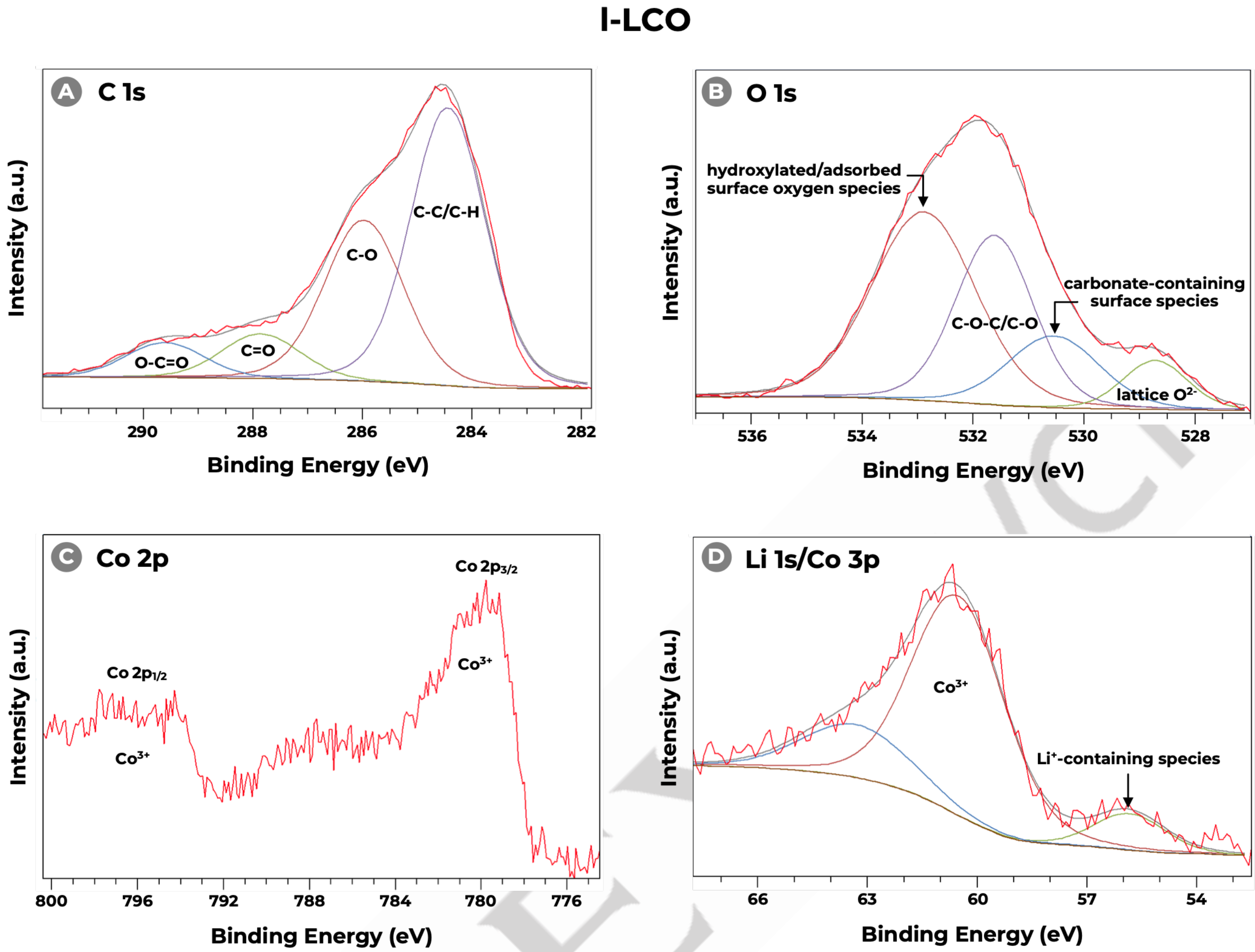

Figure S4. XPS spectra of I-LCO catalyst.

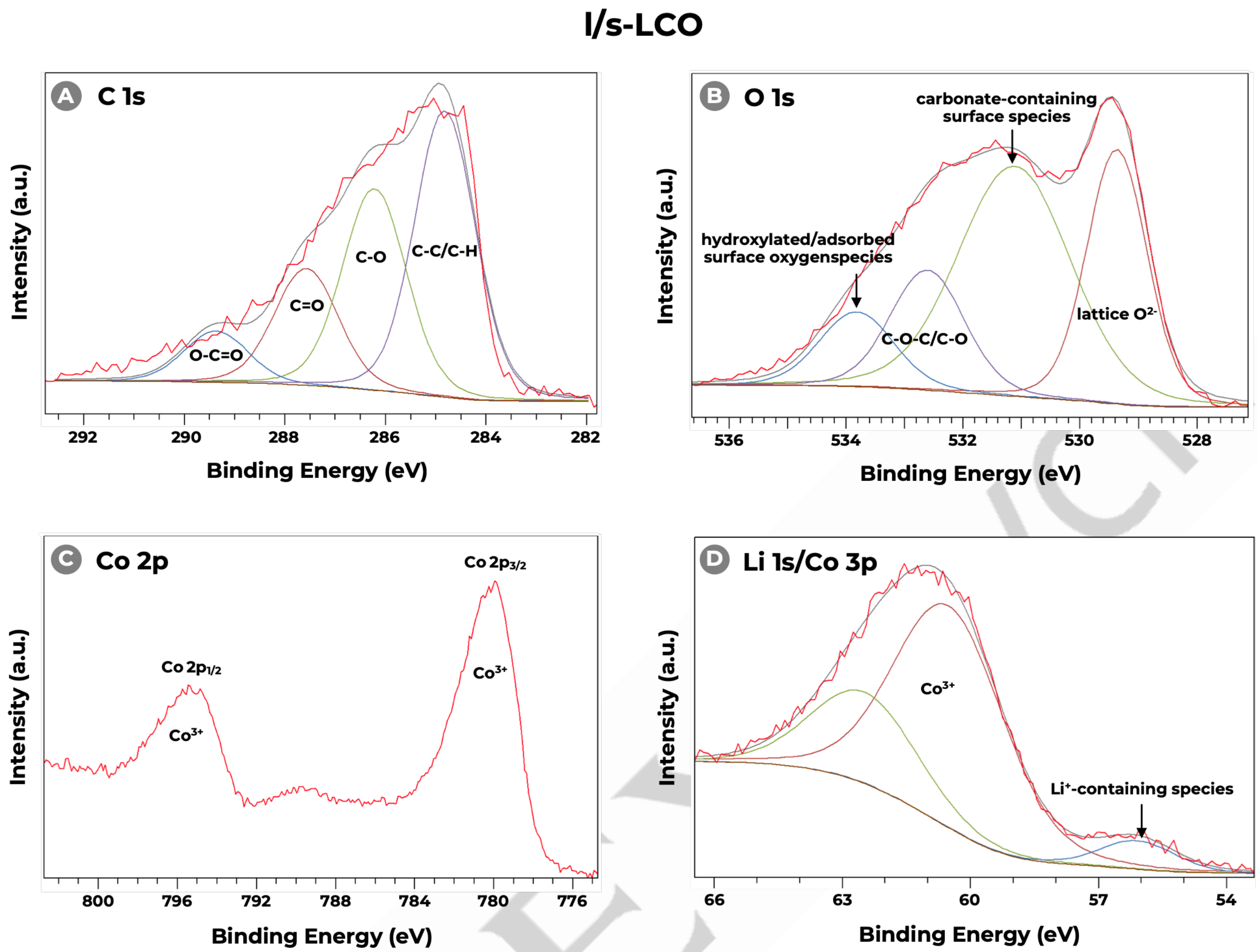

Figure S5. XPS spectra of l/s-LCO catalyst.

WILEY-VCH
